# Comparison of Fluid Flow Rates by Fluid Height and Catheter Size in Normal and Hypertensive Blood-Pressure Scenarios

**DOI:** 10.3390/healthcare12232445

**Published:** 2024-12-04

**Authors:** Nayoung Kim, Hanna Lee, Jeongwon Han

**Affiliations:** 1 College of Nursing Science, Kyung Hee University, 26, Kyunghee-daero, Dongdaemun-gu, Seoul 02447, Republic of Korea; ok3758@naver.com; 2 Department of Nursing, Gangneung-Wonju National University, Gangneung-si 25457, Republic of Korea; hannalee@gwnu.ac.kr

**Keywords:** flow rate, fluid dynamics, fluid therapy, infusion system, peripheral intravenous catheter

## Abstract

Objectives: This study is performed to determine the effects of fluid height, inner catheter diameter, and peripheral venous pressure on room-temperature intravenous fluid administration. Methods: We employed the Bernoulli equation, with frictional forces considered for volumetric analysis. Results: The results of this study demonstrate that infusion-set height, catheter size, fluid type, and blood pressure significantly affect flow rates. Under normotensive conditions, flow rates ranged from 58.2 to 10,743.18 cc/h, with the highest rates observed at a 1 m infusion-set height and larger catheters. Additionally, 6% hetastarch exhibited the lowest flow rates, while 0.9% normal saline showed the highest. Under hypertensive conditions, slightly higher infusion-set elevations were required for measurable flow rates, but they remained lower than those under normotensive conditions. Conclusion: This study investigates the mechanics of peripheral venous fluid therapy and provides foundational data for future nursing research on fluid management.

## 1. Introduction

Intravenous (IV) therapy is used to provide patients with fluids, medications, and nutrition and is administered to more than 90% of inpatients [1]. Studies have shown that nurses dedicate a significant portion of their time (between 17.5% and 24.8%) to direct care activities, which include medication administration, particularly through IV infusion [2,3,4]. However, precise control over the flow rate is crucial to ensure patient safety and treatment effectiveness [5]. Whereas peripheral IV fluid therapy allows for the precise and rapid administration of necessary medications to patients, excessive fluid administration can result in severe complications, such as acute respiratory distress syndrome, multiple organ failure, dilutional coagulopathy, and abdominal compartment syndrome [6]. Therefore, the prescribed fluid infusion rate must be maintained to reduce the possible side effects associated with fluid therapy and maximize the effectiveness of the medication administered [7].

Data reported in the United States indicate that approximately 55% of patient-safety incidents in healthcare organizations are related to medication errors and that errors related to the infusion rate are approximately 29.8% higher than those related to medication [8]. In this regard, many healthcare facilities and guidelines recommend that nurses conduct more frequent monitoring, verify the infusion rate frequently, and use flow-control devices as required to prevent medication errors and ensure accurate infusions [9]. However, even when flow-control devices intended to ensure consistent delivery are used, discrepancies exist between the displayed and actual infusion rates, hence eroding trust in these devices among healthcare professionals [7,10,11]. Furthermore, existing guidelines do not account for the manner by which the flow rate can be affected by various factors, such as the static fluid height [12], fluid concentration [13], and temperature [14].

Several factors affect the flow rate during peripheral IV fluid infusion, including patient age, health status, medication type, and dose [15]. Additionally, the Royal College of Nursing’s (RCN) Standards for Infusion Therapy [16] highlight that fluid flow depends on the vascular status. Factors such as the peripheral-vein location, catheter gauge, vein condition, blood-flow velocity [17], fluid container (bag, glass bottle, etc.), and fluid-hanger height [18] affect fluid flow. Studies have demonstrated the importance of vascular conditions for patient safety [19] and the contributions of factors such as peripheral-vein location, catheter size, and patient condition to safe IV access [20,21].

Based on common practice, flow-control devices are installed at either 51.2 cm (20 inches) from the insertion point or 76.8 cm (30 inches) above the midaxillary line; nonetheless, the absence of robust evidence underscores the necessity for further studies to establish consistent fluid rates. Therefore, peripheral venous fluid therapy must account for both the mechanical properties of fluids and the local venous pressure to maintain consistent fluid infusion and ensure patient safety. Previous studies have identified factors such as static fluid height, catheter gauge, and patient condition as influencing IV fluid flow. However, the interplay between these factors under different peripheral venous conditions has not been comprehensively analyzed. This study is novel in combining these variables to offer a more holistic understanding of peripheral venous fluid dynamics. Thus, this study is performed to provide foundational data for clinical-nursing fluid management by considering both the static fluid height and catheter size at various peripheral venous sites.

## 2. Materials and Methods

### 2.1. Study Design

In this study, control-volume analysis was performed to investigate the effects of fluid height and peripheral venous catheter diameter on the flow rates of various fluids during room-temperature IV infusion. Additionally, the specific peripheral venous pressure of each fluid type was considered.

### 2.2. Study Scope and System Description

**Figure 1:**
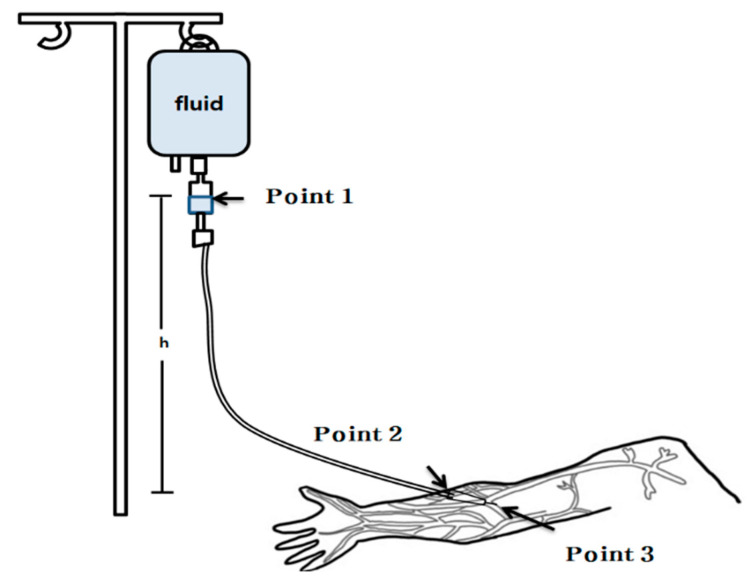
System Structure. h: Height of the infusion set is 0–100 cm; Point 1: Midpoint of the drip chamber of the infusion set; Point 2: Connection point between the infusion set and the peripheral venous catheter; Point 3: Tip of the peripheral venous catheter.

This study focused on the mechanics of an IV fluid-delivery system, specifically examining the segment from the midpoint of the drip chamber in the infusion set to the tip of the peripheral venous catheter (Figure 1).

### 2.3. Data Acquisition

#### 2.3.1. Peripheral IV Fluids

Based on the National Institute for Health and Care Excellence clinical guidelines [22], we selected commonly used peripheral IV fluids based on their suitability for various patient conditions. These fluids included 0.9% normal saline, Hartmann’s solution, plasma solution, 6% hetastarch, and 5% albumin [23,24]. The temperature of the fluids was maintained at room temperature (22 °C), as defined by the KFDA for fluids used in medical devices [25]. The viscosities and densities of these fluids had been confirmed in previous studies [13,24]. The viscosities of the fluids were 1.07 mPa/s for 0.9% normal saline with a density of 1.0046, 1.12 mPa/s for Hartmann’s solution with a density of 1.003, 1.10 mPa/s for the plasma solution with a density of 1.0064334, 2.77 mPa/s for 6% hetastarch with a density of 1.02379362, and 1.86 mPa/s for 5% albumin with a density of 1.017.

#### 2.3.2. Infusion Set

We employed a standard infusion set connected to an extension tube. The National Standard for Certified Medical Products (KSP ISO 13485:2016 [26]) defines these dimensions. The drip chamber featured a length of 64.5 mm and an inner diameter of 12.6 mm. The infusion-set tubing featured a length of 1700 mm and an inner diameter ranging from 2.6 to 2.8 mm (median 2.7 mm). An additional 900 mm extension tube was used, thus resulting in a total system length of 2600 mm.

#### 2.3.3. Peripheral Venous Catheters

We used the catheter gauges (16, 18, 20, 22, and 24) recommended by the Korean Hospital Nurses Association for adult patients [27]. The length and inner diameter specifications were based on the Korean Standards for Certified Medical Products (KSP ISO 13485:2016 [26]), which aligns with the international standards outlined by the International Organization for Standardization (ISO). The diameters and inner diameters of the catheters were as follows: 45 and 1.7 mm for 16-gauge catheters, 30 and 1.3 mm for 18-gauge catheters, 30 and 1.1 mm for 20-gauge catheters, 25 and 0.9 mm for 22-gauge catheters, and 19 and 0.7 mm for 24-gauge catheters. These specifications adhere to the guidelines set forth by KSP ISO 13485:2016 [26], reflecting the general principles and design flexibility permitted under ISO 10555 [28] and ISO 6009 [29].

#### 2.3.4. Peripheral Intravascular Pressure

Reference values from previous studies were used to account for potential variations in the peripheral vascular pressure. In healthy adults at rest and in the supine position, the peripheral vascular pressure was assumed to be 91 mmHg [30]. Novo et al. [31] categorized hypertension into essential hypertension (systolic blood pressure >140 mmHg and diastolic blood pressure >90 mmHg) and isolated systolic hypertension (systolic blood pressure >140 mmHg and diastolic blood pressure <90 mmHg) in the basilic vein. The basilic venous pressure is 200.66 mmH_2_O in adults with essential hypertension and 170.34 mmH_2_O in adults with isolated systolic hypertension [31].

#### 2.3.5. Height of Infusion Set

To satisfy the requirements of Bernoulli’s equation for steady flow, the infusion-set height was measured from the midpoint of the drip chamber to the peripheral IV access site in the supine patients. Additionally, we investigated the flow rates at various heights relative to the peripheral access point. These included heights ranging from 0 cm to the standard 100 cm, as established by the Ministry of Food and Drug Safety’s medical-device standards, with 10 cm increments between each measurement [18].

#### 2.3.6. Connection of Infusion Set to Peripheral Venous Catheter

This study incorporates the friction forces within the tubing along with Bernoulli’s equation for a more comprehensive flow analysis. The system was classified into three regions. The midpoint of the drip chamber was set as Point 1, the connection point between the infusion set and catheter was set as Point 2, and the tip of the peripheral catheter was set as Point 3. To account for friction in both the infusion set and catheter, we developed a system of Bernoulli equations with additional terms representing these frictional forces. A characteristic equation was used to model the system. Combining these equations requires a fluid-mechanics approach at the junction between the infusion set and catheter. Although the pressure remained identical at this point, owing to the physical connection, the flow velocities differed. This difference is due to the varying junction diameters, as described by the continuity equation. These velocity differences were incorporated into the Bernoulli equations along with the frictional terms to achieve a fluid-mechanically consistent model for the entire system.

### 2.4. Data Analysis

#### 2.4.1. Prerequisites

This study incorporated the frictional forces within the tubing using Bernoulli’s equation. The validity of this approach is determined by the fulfillment of specific prerequisites, such as the steady-state flow, incompressible flow, and minimal energy transfer to the surroundings [32]. First, the assumption of steady-state flow in this study refers to the overall fluid flow from the drip chamber. Whereas brief periods of unsteady flow might occur owing to the dripping pattern, they are localized and minimally affect the overall results. Therefore, the assumption of steady-state flow was considered valid for this study. Second, the fluids used were liquids at room temperature (22 °C). Compressible flow typically occurs at speeds close to the speed of sound (approximately 1500 m/s). Because these fluids are infused into peripheral veins at non-sonic speeds, compressibility can be disregarded. Therefore, the incompressibility prerequisite is satisfied. Finally, the change in fluid infusion due to the transfer between the infusion set and external energy during fluid infusion into the vessel was minimal. Therefore, the prerequisite of no external-energy transfer was satisfied in this study.

#### 2.4.2. Continuity Equations

Based on the prerequisites above, we employed the Bernoulli equation with frictional forces for analysis. The continuity equation applicable to our system is shown in Equation (1).

(1) $$A_{1}V_{1}=A_{2}V_{2}=A_{3}V_{3}$$

Subscript 1 in Equation (1) refers to the midpoint of the drip chamber, subscript 2 refers to the connection point between the end of the infusion set and the peripheral venous catheter, and subscript 3 refers to the tip of the peripheral venous catheter. $A_{1}V_{1}$ is the volumetric flow rate at the midpoint of the drip chamber, $A_{2}V_{2}$ the flow rate at the endpoint of the infusion set, and $A_{3}V_{3}$ the flow rate at the tip of the peripheral venous catheter. Based on these parameters, one can determine the fluid velocity at each point using the inner diameter of the tube.

#### 2.4.3. Bernoulli’s Equation Considering Frictional Forces

We used the Bernoulli equation for our analysis. This equation incorporates the frictional forces between designated points within the system. Equation (2) presents the Bernoulli equation with friction applied between Points 1 and 2. Similarly, Equation (3) presents the equation for friction between Points 2 and 3. By summing Equations (2) and (3), we obtain Equation (4), which represents the Bernoulli equation with friction applied across the entire system and encompasses the flow from Points 1 to 3.

(2) $$\frac{P_{1}}{\rho g}+\frac{{\alpha V}_{1}^{2}}{2g}+z_{1}=\frac{P_{2}}{\rho g}+\frac{{\alpha V}_{2}^{2}}{2g}+z_{2}+h_{f,1\sim 2}$$

(3) $$\frac{P_{2}}{\rho g}+\frac{{\alpha V}_{2}^{2}}{2g}+z_{2}=\frac{P_{3}}{\rho g}+\frac{{\alpha V}_{3}^{2}}{2g}+z_{3}+h_{f,2\sim 3}$$

(4) $$\frac{P_{1}}{\rho g}+\frac{{\alpha V}_{1}^{2}}{2g}+z_{1}=\frac{P_{3}}{\rho g}+\frac{{\alpha V}_{3}^{2}}{2g}+z_{3}+h_{f,1\sim 3}$$

#### 2.4.4. Characteristic Equations

To analyze the flow rate within peripheral vessels as a function of the infusion-set height and catheter size, we developed a characteristic equation by combining the continuity and Bernoulli equations. This equation correlates the infusion-set height, peripheral vein/catheter size, and peripheral venous pressure. The total head loss in the system represents the combined losses from the infusion set and catheter. Equation (5) shows the equation for calculating the total head loss.

(5) $$h_{f,1\sim 3}=h_{f,1\sim 2}+h_{f,2\sim 3}=f_{\alpha }\frac{L_{\alpha }}{d_{\alpha }}\frac{V_{\alpha }^{2}}{2g}+f_{\beta }\frac{L_{\beta }}{d_{\beta }}\frac{V_{\beta }^{2}}{2g}$$

Here, subscript α represents the infusion set and $f_{\alpha }$ is the Darcy friction factor of the infusion set. Subscript $L_{\alpha }$ represents the tube length, $d_{\alpha }$ the tube diameter, and $V_{\alpha }$ the average cross-sectional velocity of the fluid flowing through the tube. Subscript β represents the peripheral venous catheter and $f_{\beta }$ is the Darcy friction factor for the peripheral venous catheter. $L_{\beta }$ represents the length of the peripheral venous catheter, $d_{\beta }$ the diameter of the peripheral venous catheter, and $V_{\beta }$ the average cross-sectional velocity of the fluid flowing through the peripheral venous catheter. Whereas this study acknowledges the potential head losses occurring at the connection points between the drip chamber and tube (Point 1) and between the infusion set and catheter (Point 2), these regions represent an insignificant portion of the total flow path. Therefore, the head losses at these points, which resulted in diameter change, were considered negligible in our analysis. By disregarding these insignificant head losses in Equation (5), we can derive a simplified equation, as shown in Equation (6).

(6) $$h_{f,1\sim 3}=h_{f,1\sim 2}+h_{f,2\sim 3}=f_{\alpha }\frac{L_{\alpha }}{d_{2}}\frac{V_{2}^{2}}{2g}+f_{\beta }\frac{L_{\beta }}{d_{3}}\frac{V_{3}^{2}}{2g}$$

Substituting the expression for the Darcy friction factor ($f=\frac{64}{Re}$) in Equation (2), when the flow is laminar, Equation (6) can be obtained using Equation (7). If the flow is turbulent, then the Darcy friction factor can be calculated differently. However, in this study, the flow was assumed to be laminar. We verified the laminar assumption based on the Reynolds number.

(7) $$h_{f,1\sim 3}=h_{f,1\sim 2}+h_{f,2\sim 3}=\frac{64\mu }{{\rho V}_{2}d_{2}}\frac{L_{\alpha }}{d_{2}}\frac{V_{2}^{2}}{2g}+\frac{64\mu }{{\rho V}_{3}d_{3}}\frac{L_{\beta }}{d_{3}}\frac{V_{3}^{2}}{2g}$$

In addition, using Equation (1), which is a continuous equation, *V*_1_ can be expressed as a function of *V*_2_, as shown in Equation (8).

(8) $$V_{2}=\frac{d_{3}^{2}}{d_{2}^{2}}V_{3}$$

By substituting Equation (8) into Equation (7), $h_{f,1\sim 3}$ can be expressed as a function of *V*_3_, as shown in Equation (9).

(9) $$h_{f,1\sim 3}={kV}_{3}$$

Here, k is a constant expressed in terms of the geometric information of the drip chamber, infusion tubing, and peripheral venous catheter, as shown in Equation (10).

(10) $$k=\frac{32\mu L_{\alpha }}{\rho d_{2}^{2}g}\frac{d_{3}^{2}}{d_{2}^{2}}+\frac{32\mu L_{\beta }}{\rho d_{3}^{2}g}$$

In this study, the Bernoulli equation (Equation (4)), which accounts for frictional forces applied from the drip chamber midpoint (Point 1) to the end of the peripheral venous catheter (Point 3), can be expressed as shown in Equation (11).

(11) $$\frac{P_{1}}{\rho g}+\frac{\alpha V_{1}^{2}}{2g}+z_{1}=\frac{P_{3}}{\rho g}+\frac{\alpha V_{3}^{2}}{2g}+z_{3}+{kV}_{3}$$

The pressure at Point 1 in the drip chamber was assumed to be near vacuum, similar to the atmospheric isolation. Hence, $P_{1}\approx 0$. If the location at Point 3, i.e., the end of the peripheral venous catheter, is considered the reference for measuring height, then $z_{3}=0$. Here, *h* represents the vertical distance between the midpoint of the drip chamber and the peripheral venous catheter. The continuity equation shown in Equation (1) can be expressed as V12=(d34d14)V32. Therefore, Equation (11) is expressed in the form of a quadratic equation encompassing $V_{3}$, as shown in Equation (12).

(12) $$\frac{1}{g}\left(\frac{d_{3}^{4}}{d_{1}^{4}}-1\right)V_{3}^{2}-{kV}_{3}+\left(h-\frac{P_{3}}{pg}\right)=0$$

In this study, we utilized Equation (12) to analyze the flow rate of the fluid and interpret the relationships among the geometrical shapes of the infusion set and peripheral venous catheter, the internal pressure of the peripheral venous vessel ($P_{3}$), and the height from the end of the peripheral venous catheter to the midpoint of the drip chamber (h).

#### 2.4.5. Reynolds Number

In this study, we calculated the Reynolds number to validate the laminar-flow assumption.

(13) $$Re=\frac{\rho VD}{\mu }$$

## 3. Results

### 3.1. Analysis of Fluid Flow Rates Based on Infusion-Set Height and Peripheral Venous Catheter Size Under Normotensive Condition

**Figure 2:**
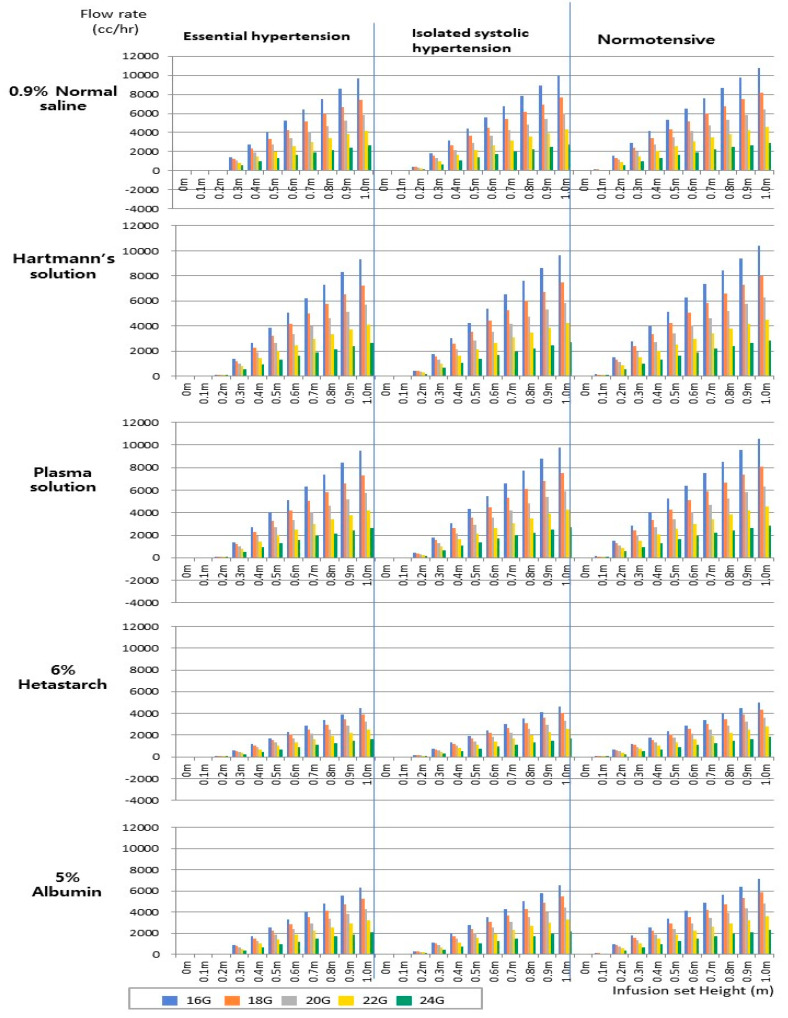
Analysis of flow rate based on infusion set height by peripheral venous catheter size in blood-pressure type.

Table 1 and Figure 2 show the relationship among the flow rates in the basilic vein, fluid height, and catheter size. Notably, areas with negative flow rates were excluded from our analysis. We observed different flow-rate ranges for the infused fluids: 58.2–10,743.18 cc/h for 0.9% normal saline, 54.2–10,400.01 cc/h for Hartmann’s solution, 57.70–10,557.72 cc/h for the plasma solution, 27.45–5032.29 cc/h for 6% hetastarch, and 38.29–7110.75 cc/h for 5% albumin. Across all fluids, the minimum flow rate occurred at a height of 0.1 m, whereas the maximum flow rate was achieved at 1 m. Additionally, for a specified height and catheter size, 6% hetastarch exhibited the lowest flow rate, followed by 5% albumin, Hartmann’s solution, the plasma solution, and 0.9% normal saline. Thus, as the infusion-set height and peripheral catheter size increased and the fluid viscosity decreased, the flow rate increased.

### 3.2. Analysis of Fluid Flow Rates by Infusion-Set Height and Peripheral Venous Catheter Size Under Hypertensive Condition

Table 1 and Figure 2 present the flow rates of various fluids in the basilic vein under hypertensive conditions. These flow rates were affected by the infusion-set height and catheter size. Notably, as with the previous study, areas with negative flow rates were excluded from the analysis. For essential hypertension, the observed flow rates were 1.7–9644.74 cc/h for 0.9% normal saline, 3.4–9324.61 cc/h for Hartmann’s solution, 3.84–9474.83 cc/h for the plasma solution, 9.91–4462.15 cc/h for 6% hetastarch, and 9.87–6327.75 cc/h for 5% albumin. For isolated systolic hypertension, the flow rates were 180.7–9952.14 cc/h for 0.9% normal saline, 171.70–9625.41 cc/h for Hartmann’s solution, 178.23–9777.79 cc/h for the plasma solution, 82.40–4620.29 cc/h for 6% hetastarch, and 116.50–6545.58 cc/h for 5% albumin. Compared with the case under normal pressure, the measurable flow rate under hypertension required a slightly higher infusion-set elevation (0.2 m). Even with increased heights and larger catheters, the flow rates remained under hypertension compared with the normal blood pressure.

### 3.3. Validity of Assumption of Laminar Fluid Flow

To assess the validity of the laminar-flow assumption, we calculated the Reynolds numbers for different fluids in the basilic vein under both normal and hypertensive conditions. These calculations were performed for two segments: the midpoint of the drip chamber to the end of the infusion set, and from the end of the infusion set to the tip of the peripheral venous catheter. In both segments, consistently remained below 2100, regardless of the fluid or pressure. This confirms that the flow within these segments exhibited laminar characteristics.

## 4. Discussion

This study investigated the effects of catheter size, infusion-set height, fluid type, and blood pressure on the flow rates in the peripheral veins of normotensive and hypertensive adults. We employed a modified Bernoulli equation that incorporated frictional forces.

Our results aligned with those of previous studies [33,34] that demonstrated a positive correlation between flow rate and peripheral venous catheter size. This is true when the fluid type, peripheral venous pressure, and infusion-set height were constant. The established clinical practice of using larger-bore catheters (16–18-gauge) for rapid fluid resuscitation, trauma patients, and contrast-enhanced imaging [35,36] aligns with the principles derived from our modified Bernoulli equation. Increasing the catheter diameter is an effective strategy for enhancing the fluid flow rate. Incorporating this principle with concrete evidence, such as our findings, into clinical guidelines can significantly improve the understanding of fluid therapy among nursing students and new graduates. Similar to previous studies [12,18], we observed a direct relationship between the infusion-set height and flow rate, based on the assumption of fixed fluid type, peripheral pressure, and catheter size. Clinical observations suggest that nurses position flow-control devices at specific heights, such as 51.2 cm (20 inches) above the insertion site or 76.8 cm (30 inches) above the midaxillary line [18]. Notably, these practices are not standardized, thus hindering accurate comparisons across different settings.

Our findings highlight the effect of infusion-set height on flow rate. The absence of specific recommendations regarding this aspect limits the ability to ensure precise fluid delivery for effective therapy. Additionally, this study revealed that increasing the height exerted a less pronounced effect on the flow rates of 6% hetastarch and 5% albumin compared with other fluids. This suggests that clinicians may have to consider increasing the infusion set beyond standard heights when administering highly viscous solutions to achieve the desired flow rates.

Our findings confirmed that flow rate is inversely proportional to fluid viscosity, based on the assumption of fixed constant pressure, catheter size, and infusion-set height. This aligns with the results of previous studies [13] that demonstrated a decrease in the flow rate with increasing fluid concentration. This relationship can be explained using the modified Bernoulli equation, where viscosity serves as a frictional force that hinders flow. Several studies [7,11] recommend using infusion pumps for highly viscous fluids to ensure accurate administration. Lower flow rates were observed for 6% hetastarch and 5% albumin, which had higher viscosities than the other fluids.

From the perspective of fluid mechanics, viscosity creates resistance to flow, which translates into lower flow rates for more viscous fluids. This aligns with Newton’s law of viscosity. Clinicians can address the challenges posed by high viscosity by increasing the catheter diameter and infusion-set height or selecting lower-pressure peripheral veins. Additionally, packed red blood cells exhibit high viscosity because of their high hematocrit level. This can hinder the achievement of the desired transfusion rate. Based on the Ministry of Health and Welfare’s Transfusion Guideline [37], diluting blood with 0.9% normal saline reduces its viscosity and facilitates faster transfusions. In general, the viscosity of a fluid decreases with increasing viscosity, although some studies [38] demonstrated that highly viscous liquids in sealed superhydrophobic capillaries flow more than ten times faster than less viscous liquids owing to gravity. This anomalous enhancement in viscosity-driven flow has been reported to be caused by viscous dampening transformations at the droplet–air interface and hydrodynamic and aerodynamic coupling with air trapped within micro/nanostructures (plastron). Hence, apart from reducing contact area and friction, plastron controls fluid flow. Therefore, the mixing of blood, fluids, and medications, which was not considered in this study, may have affected the viscosity of the mixture. Additionally, drugs administered prior to fluid therapy may have affected the flow rate. Further research regarding this topic is required.

Additionally, this study shows that a higher peripheral venous pressure resulted in decreased flow rates, based on the assumption of fixed fluid type, infusion-set height, and catheter size. This aligns with the results of previous studies, thus suggesting that the vein location and condition can affect the flow rate [17]. For instance, clinical guidelines from the Hospital Nurses Association [27] recommend avoiding lower-extremity veins for peripheral line insertion because of factors such as high venous pressure and distance from the heart, which can reduce flow and cause complications [36,39,40,41]. Our findings in patients with hypertension further support this hypothesis. An increase in venous pressure, as compared with the case of patients with normal blood pressure, resulted in lower flow rates. This aligns with the Ministry of Health and Welfare’s [39] clinical trials on fluid-therapy accuracy, which indicated flow reductions of 17% and 31% at venous pressures of 15 and 30 cmH_2_O, respectively, even when flow-control devices were utilized.

These observations highlight the importance of considering elevated venous pressure during fluid therapy in patients with hypertension. Additionally, vascular resistance and complications such as arteriosclerosis and narrowing can affect flow patterns owing to their effect on the vessel diameter. The modified Bernoulli equation suggests that the tube diameter is inversely proportional to the frictional resistance and directly proportional to the average fluid velocity. Irregularities such as hardening and narrowing can induce turbulence and further alter the flow speed. Because nurses encounter patients with various vascular diseases, they must recognize that changes in peripheral venous pressure can affect flow rates. However, the current guidelines lack specific recommendations regarding fluid-flow variations based on the underlying diseases. Integrating these findings into clinical guidelines can help nurses manage fluid therapy more precisely in patients with peripheral vascular diseases, thus ultimately ensuring more accurate fluid administration.

The findings of this study provide important insights into the factors influencing intravenous (IV) fluid flow rates, including catheter size, infusion set height, fluid viscosity, and peripheral venous pressure, with significant implications for clinical nursing practice. First, the result showing that larger catheter diameters lead to increased flow rates underscores the need for prioritizing larger-gauge catheters (e.g., 16–18 gauge) over smaller ones (e.g., 22–24 gauge) in scenarios requiring rapid fluid delivery, such as trauma resuscitation or contrast infusion during radiological procedures. This approach can optimize patient outcomes by ensuring timely and efficient fluid administration.

Additionally, the finding that increasing the infusion-set height significantly enhances flow rates highlights the importance of proper adjustment of fluid-hanger heights by clinical nurses. For highly viscous fluids such as 6% hetastarch or 5% albumin, adjusting the height appropriately or using infusion pumps can help maintain consistent and accurate delivery. Incorporating standardized guidelines that specify minimum and maximum fluid-hanger heights into clinical protocols would further enable accurate and efficient fluid management.

Highly viscous fluids tend to have reduced flow rates, as observed in this study. For fluids such as packed red blood cells, dilution with 0.9% normal saline can effectively reduce viscosity, facilitating the achievement of desired flow rates. This underscores the need for nurses to understand the relationship between fluid viscosity and flow rates and to apply this knowledge when selecting appropriate techniques to optimize fluid delivery.

Finally, the study’s finding that higher peripheral venous pressure results in decreased flow rates underscores the need for tailored approaches to IV fluid administration in hypertensive patients or those with elevated venous pressure. Using larger catheters and optimizing the infusion-set height can mitigate the effects of increased venous pressure on flow rates. This finding highlights the importance of designing fluid management strategies that take into account patients’ vascular conditions, particularly in cases of hypertension, where elevated venous pressure and vascular resistance can impede fluid delivery. In conclusion, this study provides evidence-based recommendations that can guide nurses in adopting more scientific and systematic approaches to IV fluid management. By integrating these findings into clinical practice, nurses can enhance the safety and effectiveness of fluid therapy, ultimately improving patient outcomes.

## 5. Summary of Recommendations

Based on this study’s findings, we propose the following recommendations to improve IV fluid therapy: Develop clinical nursing guidelines that include detailed specifications on catheter gauge selection, fluid-hanger height adjustments, and strategies for managing high-viscosity fluids. Introduce training programs for nursing students and new nurses focused on the interplay of physical and patient-specific factors affecting IV fluid dynamics. Conduct further research on the effects of mixing fluids or medications on viscosity and flow rates, particularly in specialized cases like blood transfusions. Establish protocols for hypertensive patients that account for peripheral venous pressure and its impact on fluid therapy outcomes.

## 6. Conclusions

This study, which is based on the mechanics of peripheral fluid therapy, aims to educate nurses on the critical factors for precise fluid infusion. Our findings can facilitate the development of evidence-based nursing guidelines and ultimately improve the efficiency of fluid management. Additionally, the analysis of flow rates in hypertensive patients conducted in this study can help nurses customize fluid therapy for accurate administration based on patient conditions.

However, several limitations should be noted. First, the study does not examine changes in fluid flow rates due to variations in intravascular pressure caused by central venous access or various peripheral vascular diseases. Second, as an in vitro study, the experimental setup utilized pressures reflective of resting and supine adults, which may not accurately represent fluid dynamics in other body positions or under dynamic physiological conditions. Additionally, the study does not address potential influences from factors such as diabetes, hyperlipidemia, or mixed fluid administration.

Future studies should expand on these findings by analyzing flow rates across different body positions and exploring the effects of various systemic conditions, such as venous diseases, diabetes, and hyperlipidemia, on fluid dynamics. Incorporating in vivo studies would allow for a more comprehensive understanding of the interactions between physiological variables and fluid flow. Furthermore, developing advanced simulation models that include these variables could help refine evidence-based nursing guidelines and improve patient outcomes in diverse clinical settings.

## Figures and Tables

**Table 1:** Flow rate based on infusion-set height by peripheral venous catheter size in blood-pressure type.

Types of IV Fluids	Height (m)	Normotensive Adults					Hypertensive Adults									
							Essential Hypertension					Isolated Systolic Hypertension				
		16G	18G	20G	22G	24G	16G	18G	20G	22G	24G	16G	18G	20G	22G	24G
0.9% Normal Saline	0	-	-	-	-	-	-	-	-	-	-	-	-	-	-	-
	0.1	137.6	125.4	107.4	85.5	58.2	-	-	-	-	-	-	-	-	-	-
	0.2	1549.5	1357.6	1131.9	872.4	577.2	3.8	3.5	3.0	2.4	1.7	441.6	398.8	339.1	267.8	180.7
	0.3	2882.6	2450.0	2007.8	1517.9	990.2	1423.7	1251.3	1045.3	807.5	535.2	1835.8	1597.2	1326.1	1017.2	670.6
	0.4	4148.7	3441.6	2785.6	2078.5	1343.7	2763.4	2354.6	1932.2	1462.8	955.3	3153.9	2665.8	2178.3	1641.6	1068.5
	0.5	5356.9	4356.0	3492.3	2580.9	1657.8	4035.2	3354.3	2717.6	2029.8	1313.2	4407.2	3639.6	2939.4	2188.3	1412.5
	0.6	6514.7	5208.9	4144.4	3040.2	1943.4	5248.5	4275.0	3430.0	2536.8	1630.4	5604.3	4540.0	3633.5	2680.6	1719.9
	0.7	7627.8	6011.2	4753.0	3465.8	2207.0	6410.6	5133.1	4086.7	2999.6	1918.2	6752.2	5381.4	4275.7	3132.2	2000.4
	0.8	8701.0	6771.0	5325.7	3864.3	2453.0	7527.6	5939.6	4698.9	3428.0	2183.6	7856.5	6174.2	4876.1	3551.6	2260.0
	0.9	9738.3	7494.4	5868.2	4240.2	2684.6	8604.3	6703.0	5274.6	3828.8	2431.1	8921.9	6925.9	5442.1	3945.0	2502.8
	1.0	10,743.2	8186.2	6384.9	4597.0	2904.0	9644.7	7429.5	5819.7	4206.6	2663.9	9952.1	7642.3	5978.8	4316.7	2731.6
Hartmann’s Solution	0	-	-	-	-	-	-	-	-	-	-	-	-	-	-	-
	0.1	129.27	117.90	100.94	80.46	54.72	-	-	-	-	-	-	-	-	-	-
	0.2	1480.43	1301.55	1087.45	840.27	557.02	7.9	7.2	6.2	5.0	3.4	417.90	377.90	321.57	254.26	171.70
	0.3	2762.00	2359.81	1939.36	1470.64	961.47	1357.46	1197.06	1002.00	775.97	515.28	1753.59	1531.67	1274.68	980.40	647.64
	0.4	3983.72	3325.68	2700.21	2021.31	1309.62	2645.07	2265.36	1864.20	1415.69	926.50	3021.92	2568.43	2104.86	1591.25	1038.05
	0.5	5153.28	4219.82	3394.15	2516.53	1619.97	3872.03	3238.84	2632.33	1972.54	1278.93	4232.17	3517.88	2850.13	2128.79	1377.16
	0.6	6276.84	5056.18	4036.23	2970.30	1902.68	5046.18	4139.01	3331.78	2472.24	1592.30	5391.66	4398.96	3532.20	2614.42	1681.07
	0.7	7359.44	5844.68	4636.59	3391.55	2164.03	6173.80	4980.28	3978.21	2929.44	1877.28	6506.27	5224.60	4164.84	3060.77	1958.89
	0.8	8405.24	6592.72	5202.44	3786.41	2408.25	7260.03	5772.91	4582.12	3353.44	2140.42	7580.86	6004.11	4757.47	3476.07	2216.36
	0.9	9417.77	7305.94	5739.12	4159.30	2638.30	8309.11	6524.46	5150.94	3750.56	2386.09	8619.44	6744.45	5316.83	3866.02	2457.40
	1.0	10,400.01	7988.81	6250.73	4513.52	2856.41	9324.61	7240.73	5690.16	4125.34	2617.37	9625.41	7451.00	5847.97	4234.76	2684.80
Plasma Solution	0	-	-	-	-	-	-	-	-	-	-	-	-	-	-	-
	0.1	136.45	124.41	106.48	84.86	57.70	-	-	-	-	-	-	-	-	-	-
	0.2	1514.69	1329.51	1109.70	856.46	567.25	8.94	8.18	7.02	5.62	3.84	434.92	392.98	334.23	264.08	178.23
	0.3	2819.29	2402.98	1972.27	1493.47	975.40	1394.40	1227.59	1026.51	793.98	526.74	1796.53	1566.18	1301.93	1000.05	659.97
	0.4	4060.92	3380.37	2740.67	2048.54	1325.90	2705.12	2311.13	1899.32	1440.25	941.58	3087.00	2616.96	2141.68	1616.68	1053.54
	0.5	5247.91	4283.63	3440.31	2546.95	1637.92	3952.02	3296.10	2674.92	2001.39	1296.27	4316.44	3577.14	2893.84	2158.14	1394.71
	0.6	6386.89	5127.42	4086.91	3003.16	1921.88	5143.61	4205.31	3379.99	2504.18	1611.23	5492.76	4466.76	3581.17	2646.65	1700.09
	0.7	7483.25	5922.15	4690.95	3426.36	2184.22	6286.66	5053.96	4030.85	2963.74	1897.40	6622.31	5299.41	4218.01	3095.23	1979.03
	0.8	8541.46	6675.49	5259.88	3822.83	2429.24	7386.65	5852.74	4638.36	3389.62	2161.48	7710.25	6084.82	4814.07	3512.33	2237.40
	0.9	9565.23	7393.31	5799.18	4197.07	2659.97	8448.11	6609.53	5210.19	3788.28	2407.91	8760.88	6830.20	5376.33	3903.76	2479.17
	1.0	10,557.72	8080.21	6313.06	4552.45	2878.65	9474.83	7330.33	5751.96	4164.36	2639.82	9777.79	7541.13	5909.93	4273.76	2707.19
6% Hetastarch	0	-	-	-	-	-	-	-	-	-	-	-	-	-	-	-
	0.1	64.09	58.64	50.32	40.24	27.45	-	-	-	-	-	-	-	-	-	-
	0.2	637.35	578.95	494.22	392.43	265.98	23.11	21.15	18.16	14.53	9.91	193.58	176.81	151.54	120.98	82.40
	0.3	1204.93	1087.12	923.63	728.77	491.28	596.77	542.36	463.15	367.94	249.48	765.54	694.31	592.06	469.41	317.75
	0.4	1767.02	1583.95	1339.87	1051.22	705.32	1164.76	1051.36	893.54	705.33	475.65	1331.88	1199.87	1018.39	802.47	540.36
	0.5	2323.76	2070.19	1744.09	1361.37	909.64	1727.23	1548.98	1310.68	1028.71	690.43	1892.75	1694.26	1431.83	1122.02	752.09
	0.6	2875.30	2546.46	2137.26	1660.52	1105.45	2284.34	2035.94	1715.72	1339.69	895.40	2448.31	2178.20	1833.48	1429.59	954.40
	0.7	3421.80	3013.38	2520.25	1949.78	1293.75	2836.25	2512.91	2109.65	1639.59	1091.79	2998.70	2652.32	2224.29	1726.44	1148.44
	0.8	3963.37	3471.46	2893.82	2230.07	1475.32	3383.10	2980.47	2493.34	1929.52	1280.59	3544.08	3117.20	2605.10	2013.60	1335.16
	0.9	4500.16	3921.18	3258.62	2502.16	1650.85	3925.02	3439.16	2867.55	2210.42	1462.61	4084.56	3573.36	2976.64	2291.98	1515.32
	1.0	5032.29	4363.00	3615.24	2766.75	1820.90	4462.15	3889.47	3232.95	2483.07	1638.55	4620.29	4021.27	3339.55	2562.33	1689.57
5% Albumin	0	-	-	-	-	-	-	-	-	-	-	-	-	-	-	-
	0.1	89.70	81.99	70.31	56.18	38.29	-	-	-	-	-	-	-	-	-	-
	0.2	931.78	838.97	711.82	560.63	377.36	23.01	21.06	18.08	14.46	9.87	276.38	251.77	215.36	171.47	116.50
	0.3	1756.21	1559.94	1311.71	1021.46	681.24	866.53	781.09	663.22	522.87	352.24	1114.49	1000.37	846.97	665.27	446.79
	0.4	2564.03	2249.58	1877.17	1448.34	959.03	1692.29	1504.69	1266.08	986.71	658.48	1935.18	1714.08	1438.76	1117.94	744.30
	0.5	3356.23	2911.67	2413.51	1847.82	1216.49	2501.38	2196.64	1834.02	1415.98	938.08	2739.49	2397.37	1997.41	1538.31	1017.21
	0.6	4133.67	3549.25	2924.83	2224.59	1457.51	3294.77	2860.77	2372.48	1817.42	1196.97	3528.37	3053.83	2527.94	1932.45	1270.77
	0.7	4897.14	4164.87	3414.33	2582.13	1684.90	4073.33	3500.17	2885.63	2195.84	1439.17	4302.67	3686.38	3034.21	2304.73	1508.58
	0.8	5647.38	4760.65	3884.58	2923.12	1900.73	4837.87	4117.43	3376.74	2554.78	1667.54	5063.17	4297.47	3519.27	2658.43	1733.27
	0.9	6385.04	5338.40	4337.69	3249.65	2106.61	5589.12	4714.70	3848.41	2896.98	1884.22	5810.59	4889.14	3985.59	2996.07	1946.79
	1.0	7110.75	5899.65	4775.41	3563.44	2303.79	6327.75	5293.79	4302.80	3224.58	2090.82	6545.58	5463.14	4435.18	3319.67	2150.66

## Data Availability

The datasets used and/or analyzed during the current study are available from the corresponding author on reasonable request.
